# Neurophysiological evidence of single-shot semantic mapping in the developing brain

**DOI:** 10.3389/fnhum.2025.1533833

**Published:** 2025-07-30

**Authors:** Marina J. Vasilyeva, Veronika M. Knyazeva, Elena D. Artemenko, Elena A. Vershinina, Ekaterina S. Garbaruk, Maria Yu Boboshko, Aleksander A. Aleksandrov, Yury Shtyrov

**Affiliations:** ^1^Department of Higher Nervous Activity and Psychophysiology, Faculty of Biology, Saint Petersburg State University, Saint Petersburg, Russia; ^2^Social and Cognitive Informatics Laboratory, National Research University Higher School of Economics, Saint Petersburg, Russia; ^3^Laboratory of Information Technologies and Mathematical Simulation, Pavlov Institute of Physiology, Russian Academy of Sciences, Saint Petersburg, Russia; ^4^Scientific Research Center, Saint Petersburg State Pediatric Medical University, Saint Petersburg, Russia; ^5^Laboratory of Hearing and Speech, Pavlov First Saint-Petersburg State Medical University, Saint Petersburg, Russia; ^6^Center of Functionally Integrative Neuroscience (CFIN), Department of Clinical Medicine, Aarhus University, Aarhus, Denmark; ^7^Cognitive Health and Intelligence Centre (CHIC), Institute for Cognitive Neuroscience, HSE University, Moscow, Russia

**Keywords:** developing brain, event-related potentials, language learning, fast mapping, semantic, word

## Abstract

Rapid acquisition of new words and construction of large vocabularies is a unique capacity of developing human brain. This process is to a large degree mediated by a neurocognitive mechanism known as «fast mapping» (FM) which allows the child to quickly map new words onto neural representations after even a single exposure to them, using context-driven inference. However, the neurophysiological bases of this mechanism are still poorly understood. To address this open question, we used event-related potentials (ERPs) to investigate brain dynamics elicited by novel words following a single-shot audiovisual semantic learning task and to estimate cortical underpinnings of this process in healthy preschool children. We found that a single presentation of novel words in association with novel objects leads to a decrease in the brain’s activation, registered as an early N400 effect for newly learnt word forms, indicating rapid lexicosemantic memory trace formation in the developing brain. Interestingly, source analysis indicated this effect to be chiefly underpinned by activity modulations in the right-hemispheric temporal cortices, indicating their involvement in speech processing at an early age (known to be diminished later in life). Overall, current findings provide the electrophysiological evidence of the specific mechanism in the developing brain that promotes rapid integration of novel word representations into neocortical lexicosemantic networks after a single exposure, subserving efficient native word acquisition and mastering the mother tongue.

## 1 Introduction

### 1.1 Fast mapping—a special form of word learning in the developing brain

The human brain has a unique capacity for rapid acquisition of large amounts of new vocabulary throughout the entire life. In fact, the process of word learning starts very early. Recent studies have shown that foundations for language learning are laid already in utero, when prenatal experiences influencing an infant’s auditory discrimination abilities subserve fetal auditory learning (Draganova et al., 2007; Partanen et al., 2013). Generally, during the course of language acquisition, children need to organize and process language structure at the multiple interrelated levels of phonology, morphology, syntax, semantics, and pragmatics. From birth and over the course of their first few months, infants display a strong preference for natural speech, and develop the ability to segment the continuous stream of speech sounds into separate words, using sequential statistical information (Saffran et al., 1996). Moreover, young infants can discriminate a great variety of phonetic contrasts, including those that are not functional in their mother tongue, but between 6 and 12 months of age they gradually restrict this ability to native language through a process of *«perceptual narrowing»* (Werker and Tees, 1984), suggesting a neural commitment to native language phonemes (Kuhl, 2004). Thus, perceptual reorganization within the first year of life results in improved discrimination of native perceptual categories and decreased discrimination of non-native ones; it is generally accepted that native language perceptual categories for vowels are formed between 4 and 6 months of age, and for consonants—between 10 and 12 months (Werker and Tees, 1984; Kuhl, 2004; Tsuji and Cristia, 2014; Chladkova and Paillereau, 2020). Across the second year of life native language phonemic categories become to dominate in perceptual discrimination and simultaneously guide the process of vocabulary acquisition (Swingley, 2009; Curtin and Werker, 2007). Perceptual narrowing in infancy has been suggested to predict later vocabulary size in childhood (Tsao et al., 2004). By the end of the first year, infants already understand the meanings of a number of words and phrases and may themselves begin to produce some words. This critical developmental milestone is often used as an objective indicator for the onset of language. It is well acknowledged that infants can understand their first words as young as 5 months; between 10 and 15 months, they begin to produce their first words; at approximately 18 months, they reach the fifty-word threshold in their productive vocabularies; and around 20 months, children usually attain the hundred-word milestone. After that point, their vocabulary development proceeds extremely rapidly, commonly estimated to be at the rate of ten to twenty new words per week. This increase in the rate of word learning is referred to as the «*vocabulary spurt»* phenomenon, and it usually occurs around the age of 18 months or around the time when 50–100 words of productive vocabulary have been acquired (Ganger and Brent, 2004). Between the ages of two and four years, children continue to build their vocabulary and develop grammatical skills. During this period, new words are learned very quickly, often after only a handful of exposures (or even after just a single encounter with a novel item). This process of rapid word learning is referred to as *«fast mapping»* (Carey and Bartlett, 1978). By the end of the second year, children can combine two or three words into simple *(«telegraphic»)* sentences, indicating the beginning of syntactic development. After 36 months, the mean length of utterances increases gradually, children can form more complex sentence structures, including questions and negations, and they start to use basic grammatical structures, such as past tense verbs and plural nouns. At the age of 3-4 years children begin to understand short stories and start to engage in simple conversations, using turn-taking and responding to questions. At this period a child’s expressive vocabulary grows to about 1,500 words (Fenson et al., 1994). By the age of 5, children demonstrate significant advancements in morphosyntactic and pragmatic language skills such as production of multi-clause sentences, understanding the rules of communication, and the appropriate use of language in different contexts. At this time, their vocabulary continues to expand rapidly, reaching thousands of words. This, to a greater extent, is driven by interactive learning, i.e., by engaging in interactions with caregivers, peers, and teachers; through shared book reading, storytelling, and singing; and etc. It is generally accepted that by school age, children typically master the basics of their native language, including the sound system, complex grammatical structures, and an organized lexicon of thousands of words (Fenson et al., 1994; Ganger and Brent, 2004; Feldman, 2019).

One of the most fascinating abilities children develop during the first years of life is ultra-rapid acquisition of novel words. Though the mechanisms underlying rapid word learning are still debated (for review, see Dollaghan, 1985; Davis and Gaskell, 2009; Shtyrov et al., 2019), it has been proposed that, in childhood, the already mentioned specific neurocognitive mechanism, dubbed «fast mapping» (FM) may be the key instrument for constructing the word meanings and rapid lexicon build-up (Carey and Bartlett, 1978).^1^ In the developing brain, the FM mechanism is usually activated in everyday communicative situations that allow a child to quickly map new words onto the lexicon after even a brief exposure. It had been proposed this early rapid word learning is context-dependent and is guided by a cognitive reasoning process, a disjunctive syllogism, that drives the mapping of novel labels to novel objects through context-based inference (Halberda, 2006).

Indeed, the FM was originally demonstrated as an exclusion-based learning (Carey and Bartlett, 1978), when a novel object (semantic referent) is presented to the child in the context of familiar ones with a new verbal label introduced at the same time. The child is able to infer the meaning of the new word by excluding other, familiar ones, which promotes an association between the unfamiliar word and the novel referent.

A large number of behavioral studies posited that the memory traces of such newly formed word-object mappings could be established and maintained even after one-trial exposure to the novel item: children as young as 3 years old could demonstrate long-term retention of such single shot-formed word representations weeks and even months later (Carey and Bartlett, 1978; Markson and Bloom, 1997; Mather and Plunkett, 2009; Vlach and Sandhofer, 2012; Kalashnikova et al., 2014; but see Horst and Samuelson, 2008; Horst et al., 2010; Kucker et al., 2018).

### 1.2 Fast mapping and rapid word learning in the adult vs. developing brain

FM has been investigated in different populations and has been described not only in children but also in adults. However, despite a large number of studies conducted in this field, their results remain controversial and the existence of FM in adults is still debated (Sharon et al., 2011; Warren and Duff, 2014; Atir-Sharon et al., 2015; Merhav et al., 2015; Warren et al., 2016; Cooper et al., 2019; Gurunandan et al., 2023). The vast majority of these studies have utilized behavioral measures only, with only a few evaluating FM in the adult brain neurophysiologically. These studies were predominantly designed as a comparative analysis of two distinct cognitive strategies of word acquisition (fast mapping vs. explicit encoding) rather than focusing on the FM as such (Sharon et al., 2011; Atir-Sharon et al., 2015; Merhav et al., 2015; Shtyrov et al., 2021, 2022; Perikova et al., 2023). Crucially, in line with the conventions in neuroimaging, requiring multiple trials to achieve high signal-to-noise ratios, they utilized a traditional approach to learning, typically with a series of repetitive presentations of word-picture pairs. However, as discussed above, the original FM framework highlights the possibility of learning a single novel item in a single exposure. One recent study in adults has followed the strategy of the classical behavioral studies and investigated the FM mechanism electrophysiologically as a single-shot exclusion-based learning (Vasilyeva et al., 2019). Its results indicated that FM could promote rapid integration of the learnt novel word forms into cortical memory networks from a single exposure: a significant change in neural activation for a newly learned word was found at ~200–400 ms after semantic training and was supported by the left fronto-temporal cortical sources. These findings confirmed some previous neuroimaging studies in adults that claimed that the FM mechanism may activate hippocampally-independent route of cortical learning, inducing rapid neocortical plasticity to create novel word-object associations – even before the slower hippocampus-dependent stage of overnight consolidation takes place (Coutanche and Thompson-Schill, 2014; Atir-Sharon et al., 2015; Merhav et al., 2015; but see Gurunandan et al., 2023).

Importantly, this single-shot neural memory trace build-up in the adult brain was found for stimuli with native phonology, suggesting the engagement of pre-existing language circuits (most likely phonological ones) in mastering new words of the mother tongue (Vasilyeva et al., 2019). These results are in line with some previous ERP studies in adults, obtained with passive perceptual learning paradigms, that revealed a significant modulation of early (~50–150 ms) neural activity in fronto-temporal cortical networks, indexing rapid learning of novel phonologically native-like word forms (Shtyrov et al., 2010; Shtyrov, 2011; Kimppa et al., 2015). Crucially, these adult studies found no learning effects for either phonologically non-native word forms (Kimppa et al., 2015) or acoustically matched non-speech sounds (Shtyrov, 2011). In contrast to adults, similar mass passive exposure of school-age children to word forms with both native and non-native phonology as well as to non-speech sounds led to rapid activity changes taking place over just a few minutes (Partanen et al., 2017), indicating an enhanced plastic capacity of the developing brain. Furthermore, these plastic changes were underpinned by activity distributed across multiple areas of both left and right hemispheres, demonstrating large-scale cortical networks involved in the formation of memory traces for new auditory patterns in young age (unlike the left-lateralized effects in adults). Notably, such a participant-friendly passive learning paradigm that relies on multiple (dozens or even hundreds) repetitions of novel items does not typically involve any meaning assigned to them, which makes it problematic for studying the semantic component of word acquisition.

### 1.3 N400 component in word learning and semantic processing

Given the complex rapidly unfolding temporal dynamics of linguistic communication and the highly dynamic transient brain activity accompanying it, temporally resolved neuroimaging tools, such as electro- and magnetoencephalography (EEG, MEG) are often posited as being most suited for studying neurolinguistic processes (Grimaldi et al., 2023). Among different electrophysiological measures employed to investigate neural underpinnings of novel word meaning acquisition, the most commonly used event-related potential (ERP) is the so-called N400, a negative-going component with a typically centroparietal maximum at approximately 250–500 ms (peaking around 400 ms) post-stimulus. In children the latency and distribution of this component (and others) may be different due to the continuous maturation of brain structure and functions (e.g., the speed of information processing, memory efficiency, etc.; Atchley et al., 2006; Friederici, 2006; Friedrich and Friederici, 2011). The N400 is a sensitive index of word learning and meaning processing, linked to lexical and semantic features of verbal stimuli. Its amplitude changes as a function of a word’s meaningfulness or predictability within the surrounding semantic context: it may vary from very large when a word is used inappropriately or has no clear meaning, which makes its integration difficult, to very small when a word is quickly understood and/or can be integrated with ease. Importantly, the N400 has not only been linked to different aspects of semantic processing in sentences and larger contexts, but also to single word processing. N400 amplitude was found to be larger for pseudowords (i.e., meaningless word-like stimuli) than for real words, reflecting the difficulty in identifying lexical representations for unfamiliar word forms in the mental lexicon (for review, see Kutas and Federmeier, 2011).

Some previous studies on spoken sentence processing have generally documented that auditory N400 effect might be preceded by a negative voltage deflection with a fronto-central distribution that peaks in the 150–350 ms time window after word onset (Connolly and Phillips, 1994; Hagoort and Brown, 2000). This auditory «pre-N400» was predominantly registered for sentence-final-word phonemic violations and was sometime called phonological mismatch negativity (PMN; Connolly and Phillips, 1994; Poulton and Nieuwland, 2022). Some authors referred to this component as N200-250 (Hagoort and Brown, 2000; Van Den Brink et al., 2001) and also as «early auditory N400» (Holcomb and Neville, 1990; Van Petten et al., 1999). More generally, research data provide evidence that, in well-controlled contexts, N400 indeed starts around 200 ms (Friedrich et al., 2006), likely indicating that this earlier negative deflection is still part of the same N400 complex.

Other studies reported this early N400 effect in paradigms with isolated single words or sounds, for instance, in the phoneme deletion task (Newman et al., 2003), in different types of auditory word recognition paradigms (e.g., visual picture/spoken word matching task; see Desroches et al., 2009; Sasaki et al., 2023), and even with words that contain acoustically manipulated coarticulatory cues (Archibald and Joanisse, 2011).

The early N400 effect has also been documented in developmental studies utilizing cross-modal match/mismatch paradigm (Henderson et al., 2011; Junge et al., 2012; Junge et al., 2021). In young children, it was often considered as a part of the broader N400 effect in the 200–600 ms range (Friedrich and Friederici, 2004, 2005a, 2005b). On the other hand, some authors have also claimed that, although onsets/offsets of such broader negativity are usually hard to define, the earlier and later time frames may correspond to functionally separate processes with different developmental trajectories at early ages (Junge et al., 2012; Junge et al., 2021).

Importantly, the N400 has also proven to be a convenient tool for investigating the new word learning in children, even in pre-verbal participants. Developmental N400 studies using a word-picture match/mismatch paradigm reported that infants and young children could quickly map new words onto representations, as reflected in N400 amplitude reduction, although given at a very slow pace, with some number of training trials given at a very slow pace, with some contradictory results (Friedrich and Friederici, 2004, 2005a, 2005b, 2011). It has been suggested that, already at the early pre-verbal age, new word learning is based on rapid formation of declarative memory traces, although various limitations during consolidation stage (such as underdeveloped medial temporal lobe/hippocampus in young children; e.g. Bauer, 2008; Weiss-Croft and Baldeweg, 2015) could dramatically reduce the effect of learning and the ability to reproduce new knowledge (Friedrich and Friederici, 2008, 2011). Importantly, although such N400 studies in young children have been instrumental in uncovering neural learning process, they did not involve the classical FM paradigm in its strict sense: that of single-shot inference-driven learning.

### 1.4 N400 component in contextual word learning

Two N400 studies of contextual novel word learning reported successful results after a single presentation of a new item in visual modality to young adults (Borovsky et al., 2010, 2013). Participants were required to derive the meaning of novel word form, presented visually in one trial, from sentences that strongly or weakly constrained its meaning. The results revealed that, after a single presentation of a target new item in a sentence with a highly predictable informative context, the N400 amplitude was significantly lower when the newly learnt word was used in a plausible context in test sentences, as opposed to implausible context. The authors interpreted this rapid single-shot effect, manifest in significant N400 amplitude decrease, as a reflection of successful lexicosemantic learning in highly informative contexts. This is a very important result; yet, it still has to be noted that this paradigm is again different from the classical disjunctive syllogism-based FM approach.

Further studies with the same learning paradigm attempting to replicate these results in school age children were less encouraging (Abel et al., 2018, 2020). At least three exposures to novel word form in sentential context were needed to evoke neural signatures of semantic processing in 8–11- and 11–14-year-olds: N400 amplitudes for novel words with identified meanings became indistinguishable from those to familiar ones and differed from novel items with unidentified meanings.

### 1.5 Summary of studies and unresolved questions

In sum, previous studies revealed online changes of brain dynamics during rapid novel word learning via FM, but most of them did not follow the original design to investigate the FM learning mechanism in its strict sense. Such studies predominantly used series of paired word-picture presentations or, paradigms with multiple word form repetitions with no meaning assigned to the stimuli or specific context-restricted learning tasks. Indeed, using a one-trial presentation of novel items to study on-line formation of novel word representations seems extremely challenging in neurophysiological research. To our knowledge, only few EEG studies successfully implemented a single-shot word learning design and revealed distinct neurophysiological indices of new memory trace build-up: two operating with visual stimuli during short sentence reading (Borovsky et al., 2010, 2013), and another one performed in auditory modality (Vasilyeva et al., 2019), the latter being more natural for the language acquisition and for the evaluation of FM mechanism as a special form of word learning. These studies were performed in adults; thus, the neural underpinnings of this ultra-rapid word learning mechanism in the developing brain are still largely unknown.

Importantly, comparative studies indicated that the development of language processing change quantitatively and qualitatively across the lifespan. It is generally assumed that in the adult brain normal language function is based on functional interplay between left (LH) and right (RH) hemispheres, with well pronounced LH dominance and active (albeit more complex) involvement of the RH in various aspects of language, for example, in processing of paralinguistic features, metaphor comprehension, as well as semantics and acquisition of novel word forms (Jung-Beeman, 2005; Lindell, 2006; Federmeier et al., 2008; Paulesu et al., 2009; Vigneau et al., 2011; Passeri et al., 2015; Ulanov et al., 2023). Conversely, in children the laterization of the language function undergoes a shift during development, demonstrating enhanced RH involvement in language processing particularly at early ages (Chou et al., 2006; Lindell, 2006; Brauer and Friederici, 2007) and gradual development of LH dominance during maturation (Olulade et al., 2020; Martin et al., 2022).

However, despite numerous studies the relative involvement of the RH and LH hemispheres in the formation of word representations and acquisition of new semantics remains unclear. It was proposed that the two hemispheres differently contribute to the initial stage of semantic mapping (Beeman et al., 1994; Bowden and Beeman, 1998; Jung-Beeman, 2005), such that «fine» semantic coding is provided by LH, whereas RH, carries out a relatively «coarse» semantic coding (Beeman et al., 1994; Chiarello et al., 2003; Deacon et al., 2004; Grose-Fifer and Deacon, 2004; Jung-Beeman, 2005; Faust, 2012). Several studies have claimed that this «coarse» coding might be provided by the RH during rapid word learning, particularly after a single-shot encounter with the new item (Ince and Christman, 2002; Borovsky et al., 2013). Thus, we can hypothesize that, in the developing brain, the RH may be actively recruited during rapid FM learning, contributing at least to the initial stage of novel semantic mapping. Later on with growing experience of using the word during repeated exposures in different contexts, and strengthening of the lexicosemantic representation, the relative RH and LH contributions would be gradually re-shaped, with LH playing a more expressed role.

Furthermore, a growing body of research indicated that the child’s brain is much more malleable/susceptible to learning than that of adults and is extremely sensitive to a wide range of experiences and environmental demands, manifesting an outstanding capacity for rapid plastic changes (Galván, 2010; Benasich and Ribary, 2018; Ekerdt et al., 2020; Wenger and Kühn, 2021; Frank et al., 2022). These findings come from different domains of developmental research, and the most remarkable evidence has been documented for language, where extraordinary amount of information is acquired at a dramatically high rate (Kuhl, 2004, 2010; Saxton, 2008; Ramírez-Esparza et al., 2017; Bosseler et al., 2021). Although various studies conducted in this field revealed a large variety of outstanding results, several open questions still remain. How is new word meaning constructed by the developing brain – in particular via fast single-shot word-object mapping? Are both fundamental components of word learning – the word’s phonological form and the word’s meaning – accessible, and appropriately situated within the semantic space of the child’s mental lexicon as a result of such ultra-rapid learning? And, importantly, what are the neural correlates of single-shot word learning (in strict inference-based FM sense) in the developing brain? To our knowledge, no previous neurophysiological studies predominantly utilized proper FM paradigm in its exact sense to evaluate this specific ultra-rapid word learning mechanism in children. Given this lack of research on the neural processes underlying novel word learning through FM in children, we designed the current EEG experiment to fill these gaps.

### 1.6 Current study

To investigate the neural correlates of FM learning in the developing brain we implemented a new experimental procedure aimed to satisfy a number of criteria: (1) to model the process of new word acquisition in young children taking place in everyday naturalistic communicative situations, (2) to enable assessment of the outcomes of single-trial learning, and (3) to be adaptable to conventional EEG laboratory settings. To this end, we combined a one-trial semantic associative learning task with a short passive session of auditory ERP recording. To study how the developing brain constructs new representations for natural speech sounds, we used a counterbalanced set of audiovisual stimulus pairs, such that each type of auditorily presented item (familiar word vs. novel word form) was assigned a visually presented semantic referent (real familiar or previously unknown novel object). During the task, after a single acoustic stimulus presentation, the child had to select the visual referent defined by the word form, such that the new meaning became apparent through inference/exclusion process. Only one trial was used to present the novel item. To evaluate learning-related brain activity, we recorded, immediately after this FM learning session, passive auditory ERPs (that are well-known to reflect memory-trace activation; Shtyrov et al., 2010; Shtyrov, 2011; MacGregor et al., 2012; Kimppa et al., 2015; Partanen et al., 2017; Vasilyeva et al., 2019) elicited by both familiar and newly learned items. As a control condition, we used phonologically native familiar and novel word forms that did not undergo the present FM procedure. We hypothesized that this procedure would lead to immediate changes in ERP dynamics registered after a single-shot semantic learning task, possibly manifest as a change in N400 response amplitude for trained as opposed to untrained items, thereby indexing the formation of new representations in the developing brain.

## 2 Materials and methods

### 2.1 Participants

Twenty four children (5–7 years old) participated in the study. All participants were monolingual native Russian speakers, without neurological diagnoses, sensory or speech impairments or hereditary diseases. All children attended kindergarten.^2^ Data on the psychosomatic and neurological status of each participant were obtained from medical records provided by kindergarten medical staff. Four children were excluded from final data analysis due to low EEG recording quality, which resulted in a sample of twenty children included in the final dataset (M = 6.48 years; SD = 0.75; 6 boys). Child–parent pairs were remunerated for their participation (using toys, children’s books, etc.). Informed consent was obtained from all participants and their parents. The study was approved by the Ethics Committee of St. Petersburg Psychological Society (protocol #21 of 06.04.23) and conducted in accordance with the Helsinki Declaration.

### 2.2 Stimuli

#### 2.2.1 Acoustic stimuli

A counterbalanced set of acoustically and acoustically matched and strictly controlled stimuli was developed to be used in the learning session and the passive ERP recording afterwards. To this end, consonant-vowel (*CV*) syllables were recombined to form dissyllabic (*CVCV*) stimuli of two types: (1) meaningful phonologically native Russian words, and (2) phonotactically and phonologically legal meaningless novel word forms (pseudowords) with native phonology.

To create two stimulus types, word-initial syllables *va, si, lu, re* (IPA transcriptions: [va], [si], [ɫu], [rʲe]) and word-final syllables *ta* and *pa* (IPA: [tɐ], [pɐ]) were recorded separately, to avoid co-articulation confounds. Then, these first and second syllables were combined to form four native concrete nouns (*vata, repa, lupa, sita –* Eng.: cottonwool, turnip, magnifying glass, sieve/strainer) and four pseudowords with native phonology (*vapa, sipa, luta, reta*) of highly similar make-up, yet with no meanings.

The acoustic stimuli were selected such that their acoustic-phonetic properties could be precisely controlled, while their lexical and phonological status could be manipulated. Most importantly, the design, with counterbalanced disyllabic make-up, implied that all items could only be completely recognized from their second syllables only. This allowed us to define the *divergence point* after which the stimulus (familiar word vs. pseudoword) could be fully identified and thus the ERPs could be time-locked to. This disambiguation point was at 359 ms from the stimulus onset, when the second syllable started. The total duration of all stimuli was 535 ms.

The stimuli were produced from digital recordings (44.1 kHz, 32 bit); Adobe Audition 3.0 (Adobe Systems Inc., San Jose, CA, United States) of a female monolingual native Russian professional speaker, obtained in a soundproof chamber. Adobe Audition v. 3.0 and Praat v. 6.0.4 (Boersma and Van Heuven, 2001) software packages were used for stimulus editing. The syllables were normalized for root-mean-square (RMS) power, fundamental frequency (F0) and duration. Final syllables were also normalized by the maximal peak amplitude, consonant duration and time before the burst of the vowel. To conform to the natural stress and prosody of Russian (Potapova and Potapov, 2012), the duration of the final vowel was always shorter with F0 decreased by 5%. Finally, all acoustic stimuli were validated in a separate perceptual ranking study. To that end, six adult Russian native speakers were presented the two stimuli types with a task to report what they had heard. 100% correct identification rate was obtained, validating the intended stimulus design.

#### 2.2.2 Semantic referent stimuli

In the present study, we aimed to model the rapid process of new word acquisition that occurs in the naturalistic settings of young children’s everyday lives. Therefore, we placed special emphasis on the stimuli used to provide semantic reference for the novel sounds. To this end, rather than using on-screen images, we employed a set of real objects as visual referents corresponding to the acoustically presented familiar and novel word forms. The objects were carefully selected to be of high quality and to look as either very familiar (providing the context for exclusion/inference) or unknown (referents for novel items) to the children, so as to pique their interest and encourage their engagement with the learning task. We thoroughly controlled the quality of these stimuli and ensured that all objects were approximately the same size, varied in color and texture, and were made from different materials, such as metal, wood, rubber, plastic, or modelling clay.

Four real objects were chosen to represent familiar items and corresponded to the acoustically presented familiar native words (i.e., cottonwool (*vata*), turnip (*repa*), sieve (*sita*), magnifying glass (*lupa*)). As the unknown stimuli, two objects representing a rare technical tool and an ancient musical instrument were selected, to be paired with acoustically presented novel native word forms (one novel object was used in practice session and the other – in learning session). Note that between the two stimuli of each category, one was used only in the learning block, and the other one only as a control stimulus in the ERP recording, with the specific tokens rotated and counterbalanced across the sample. The complete list of acoustic and visual (semantic referent) stimuli is presented in the Supplementary material.

To validate the familiarity contrast, ten adults and ten child–parent pairs (separate from those in the main EEG study) participated in an independent expert evaluation of the familiar and unknown visual stimuli using an online questionnaire with color photographs of the stimuli. The results showed that the novel objects were not recognizable, while all familiar objects were well identified (see Supplementary Table 1).

### 2.3 Experimental design and procedures

**Figure 1:**
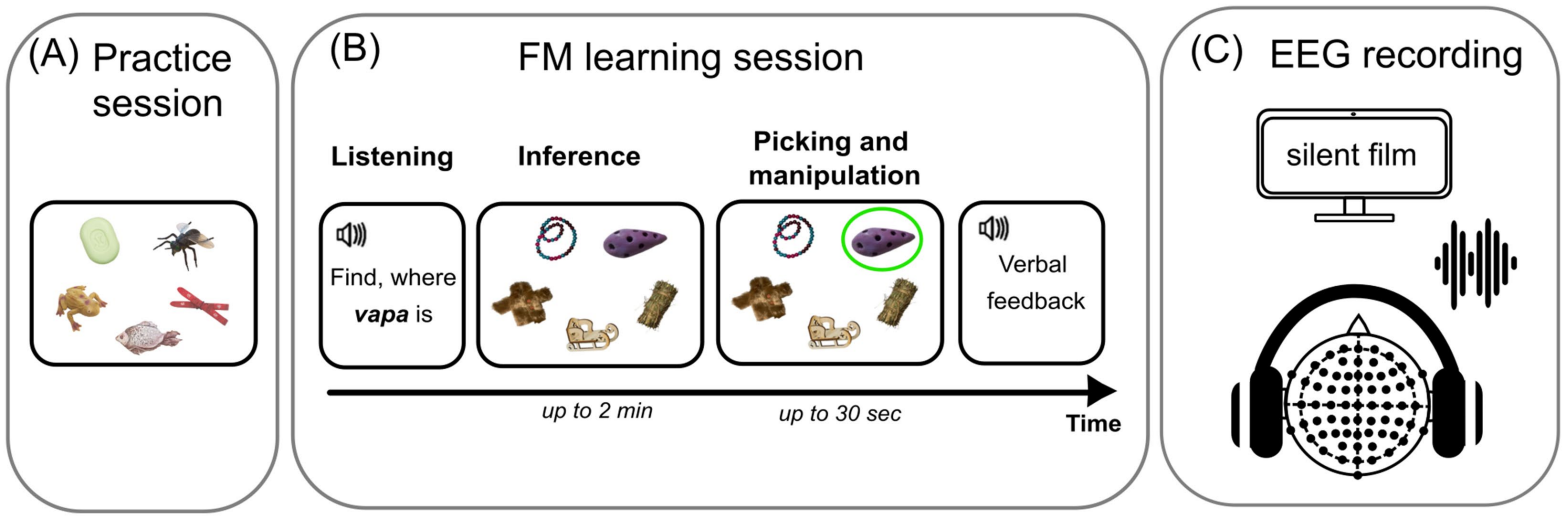
Schematic illustration of experimental procedures: practice, novel word learning (fast mapping) and EEG recording sessions. **(A)** Practice session: example of a trial with familiar word-object pair (for details see Materials and methods). **(B)** Learning session: example of the experimental sequence in a fast mapping trial with an unknown word-object pair (the unfamiliar object is highlighted by a green circle). **(C)** Passive session: passive auditory ERP recording.

Experimental design consisted of a short practice session followed by an FM learning session, immediately after which a passive ERP recording session was conducted (Figure 1).

#### 2.3.1 Practice session

Practice session was designed to familiarize the child with the task and with the testing procedures. We utilized five practice trials with familiar word-object pairs and one trial with an unknown word-object pair (using stimuli not employed in the experiment proper later). All participants successfully completed the practice session.

#### 2.3.2 FM learning session

This session was aimed at invoking the child’s ability to infer the meaning of a novel word from the semantic context through a single-shot exposure to a novel item by excluding other, familiar items/words. To achieve this, we implemented a child-friendly audio-visual learning paradigm (Figure 1). In a counterbalanced stimulus set, each auditory stimulus (presented via headphones) was assigned a semantic reference: familiar or novel object (presented visually on the tray in front of the child). This closely followed the original FM studies, with a spoken request containing the target word (e.g., “find, please, where the *vapa* is”). The child had to choose a new object defined by a novel word form; this could only be achieved by excluding other, familiar items, as no further reference or assistance was provided. Only one mapping trial was administered. If the child succeeded in referent selection, an explicit positive feedback – verbal praise – was given. As it is generally assumed that implicit learning may precede overt behavioral responses (Cleeremans et al., 1998; Gathercole, 2006; Quinton and Brunton, 2017; Broedelet et al., 2023), so the lack of correct inference during FM-session was not an exclusion criterion in the current study. There were only two such participants, with the vast majority of single-shot exposures (18 children) leading to successful inference.

In the FM session, the target object was always displayed beside the four other, familiar objects (Figure 1). This session always started with two additional familiarization trials that included only familiar word-object pairs, to ensure that the children fully understood the task and were comfortable with it. Following this, two FM trials were presented in random order: one including a familiar word-object pair and one with a target new word form and novel object. The stimuli and the sound-referent pairings were fully counterbalanced across the participants. Auditory presentation was controlled using NBS Presentation v.20.0 software (Neurobehavioral Systems, Inc., Berkeley, CA, United States).

#### 2.3.3 EEG recording (passive session)

During ERP recording, the participants were instructed to pay no attention to the acoustic stimuli and to focus on a silent video film. Both stimulus types (familiar words and novel words forms) were presented in this passive listening fashion such that half of stimuli of each type were those used in the FM session, and the other half served as a control untrained condition, making a 2 × 2 design (for details see section *2.4*); the actual tokens were fully rotated and counterbalanced across the sample. Furthermore, given the stimulus design, half of the stimuli ended with syllable [ta] and half – with [pa], while sharing their CV onsets in a counterbalanced fashion, such that each individual stimulus could be recognized only at the second syllable. Auditory stimuli were presented in two subblocks (to reduce fatigue) binaurally via headphones, with 50 trials of each stimulus in one block (totally 400 stimuli) in pseudorandom order such that the same stimulus could not be repeated two consecutive times. The stimulus onset asynchrony (SOA) was jittered randomly between 1,100 and 1,200 ms in 10 ms steps. Stimulus presentation was controlled using NBS Presentation v.20.0.

All EEG recordings were conducted in an acoustically and electrically shielded room (Neuroiconica Ltd., St. Petersburg, Russia) at the Laboratory of Behavioral Neurodynamics (St. Petersburg State University). EEG signals were recorded with 64 Ag/AgCl active scalp electrodes mounted on an elastic cap (Easy Cap GmbH, Wörthsee, Germany) according to the international 10–20 system (Jasper, 1958) and connected to an actiCHamp BrainVision EEG amplifier (Brain Products GmbH, Gilching, Germany). BrainVision PyCoder v.1.0.9 software was used to control the EEG recording, with the online reference at FCz electrode location, 1,000 Hz sampling rate, and 0.01–1,000 Hz recording bandwidth. To monitor vertical eye movements, an additional EOG electrode was placed below the right eye. Electrode impedances were kept below 25 kOm.

#### 2.3.4 Procedure

All experimental procedures were performed in the presence of the child’s parent(s). Prior to the experiment, sufficient time was given to the child and parent to become comfortable and adapt to the laboratory environment. During the experiment, the child was seated in a comfortable chair with the parent sitting adjacent to them, near a table. A tray containing the objects used in the training session and learning task was situated on the table in front of the child.

In total, three experimental blocks were administered to each participant: training session, learning session and passive EEG recording session. The duration of the training session was about 7 min; the learning session – about 4–7 min; the EEG recording session – about 8 min. The total duration of the experimental blocks was about 22 min. Short breaks were given between the experimental blocks or when the child showed any signs of fatigue.

Since, according to the FM framework, the learning-related brain dynamic should be present immediately, the EEG cap was put on right before the training session began. Thus, immediately (not more than 2–3 min) after the training, the EEG recording commenced.

During all experimental procedures, the parent was instructed to sit calmly and silently and not to help/prompt the child during the task. One of the researchers sat near the child during the training and the learning sessions to run the experiment, and to monitor the child’s comfort level and engagement in the task. During the ERP recording, the child was instructed to pay no attention to the acoustic stimuli and to focus on a silent video film. The parent was additionally instructed to sit quietly, to watch a silent video film and to monitor the child’s comfort level.

### 2.4 ERP data processing

After the recording, the EEG data were off-line processed using BrainVision Analyzer v.2.1 (Brain Products GmbH). The data were downsampled to 250 Hz and re-referenced to a common average reference computed across all EEG electrodes, and a zero-phaseshift 8-order Butterworth 0.5–50 Hz and 50 Hz notch filters were applied. Eye movements were corrected using Infomax ICA-based correction algorithm, and signals at any noisy channels were reconstructed by interpolation. Intervals containing voltage jumps exceeding ± 100 μV or differences of values over 200 μV in any 200 ms periods well as intervals containing voltage lower than 0.5 μV over 100 ms or longer, were considered artifacts and subsequently excluded from further analysis (with 200 ms before and 300 ms after artifact event discarded). Thereby cleaned EEG data were then segmented into epochs based on stimulus type (trained and untrained real and novel words), with a time interval from 100 ms pre-stimulus to 900 ms post-stimulus, and a baseline correction interval from 100 ms before stimulus onset to the disambiguation point was applied. Average number of trials that remained after EEG artifact removal was 83.7 ± 11.31 (60–99). After artifact rejection and segmentation, the data were averaged separately for each stimulus type to obtain ERP waveforms. For an unbiased data-driven identification of the main peaks of ERP activity, global field power (GFP) was computed across all electrodes for all stimuli, conditions and participants. GFP indicated the most prominent peaks at 406, 566 and 662 ms from the stimulus onset (corresponding to 47, 207, and 323 ms after the divergence point, i.e., the second syllable onset), which were analyzed further. Mean (baseline-to-peak) amplitudes were extracted from 50 ms wide time windows around these individual peaks, leading to three distinct intervals selected for the statistical analysis: 22–72, 182–232 and 298–348 ms after the stimulus divergence point. Given the stimulus design, the same stimulus type was presented by both *pa-* or *ta-*ending tokens in different participants, counterbalanced across the group. These were averaged together per stimulus type, thereby removing any possible influence of acoustic differences between participants or conditions. As maximal ERP amplitudes were recorded predominantly at fronto-central electrode sites, we focused the analysis on the electrode array covering these fronto-central channels, subdivided into left- and right-hemispheric clusters: left (FCL: F1, F3, F5, FC1, FC3, C1) and right (FCR: F2, F4, F6, FC2, FC4, C2).

To assess differences between different stimulus types and learning conditions in the ERP data, two-way repeated–measures analysis of variance (rmANOVA) with factors Stimulus Type (familiar word/novel word form) and Learning Session (untrained word form/trained word form) was used (as implemented in SPSS v. 21 software, IBM Corporation, New York, United States). Greenhouse–Geisser correction was applied whenever the sphericity assumption was violated. Window-mean ERP amplitudes were submitted to rmANOVA that was conducted separately for each cluster (FCL and FCR) and for each of the three main time windows (22–72, 182–232 and 298–348 ms).

### 2.5 Source analysis

Where a statistically significant difference was found in the ERP analyses, underlying cortical generators were estimated using low-resolution electromagnetic tomography (sLORETA; Pascual-Marqui et al., 1994) as implemented in Brainstorm software v. 3 (https://neuroimage.usc.edu/brainstorm; Tadel et al., 2011). Age-appropriate MRI templates, with average templates available for each age group with 6-month intervals (5; 5.5; 6; 6.5 and 7 years) were obtained from the Neurodevelopmental MRI Database (Richards et al., 2016). These templates were first segmented using Brainsuite software v. 19b^3^ based on BCI-DNI brain atlas (Pantazis et al., 2010) and then utilized to create boundary element models (BEM) in Brainstorm software with 1922 vertices per layer (scalp, inner and outer skull with 4 mm thickness).

For statistical analysis of source data, regions of interest (ROIs) were chosen *a priori* on the basis of previous investigations on neuroanatomical foundations of language (Hickok and Poeppel, 2007; Friederici, 2011) and consisted of established language areas in the temporal lobe (regions of the inferior, middle and superior temporal gyri), temporal pole, inferior frontal, and supramarginal areas as well as their right-hemispheric homologues. Mean current densities were extracted from these ROIs over time windows that had demonstrated statistically significant effects in the ERP analyses. To assess differences between different stimulus types and learning conditions, a similar rmANOVA as used above for the ERPs with factors Stimulus Type and Learning Session was conducted separately for each ROI. Greenhouse–Geisser correction was applied whenever the sphericity assumption was violated.

## 3 Results

### 3.1 ERP results

**Figure 2:**
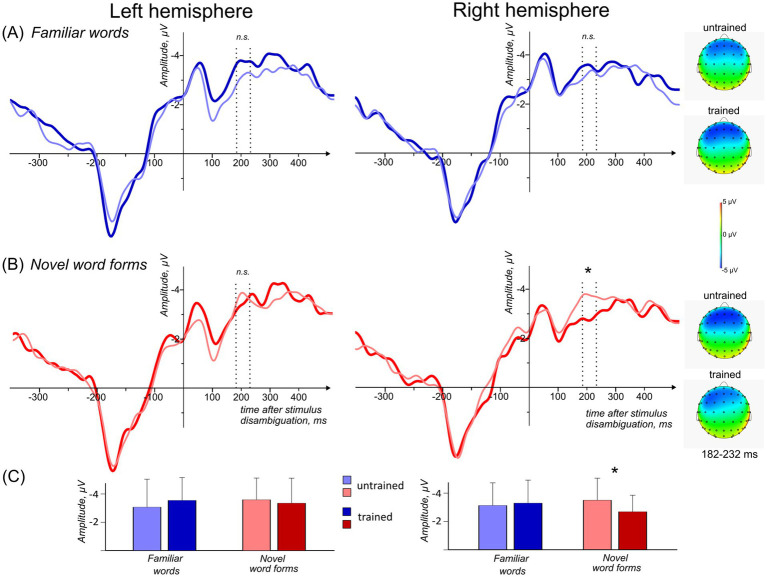
Average ERPs and mean voltage topographic scalp maps in response to familiar words and native-like novel word forms for FM-trained and untrained control conditions at the left and right fronto-central clusters. **(A)** Average ERPs and mean voltage topographic scalp maps in response to familiar words and **(B)** in response to novel word forms. Negativity is plotted up. ERPs are time-locked to the word divergence point, i.e., the critical second syllable onset after which the stimulus (familiar word vs. novel word form) could be fully identified (the word divergence point corresponds to the zero point on the y-axis). Vertical dotted lines indicate 182–232 ms time interval where significant learning-induced ERP modulation was found. Mean voltage topographic scalp maps for this window are presented on the right. **(C)** Average ERP amplitudes for 182–232 ms time window at the left and right fronto-central clusters. Error bars denote standard error of the mean (SEM); * Asterisk denotes statistical significance (*p* < 0.05); non-significant differences are marked as “n.s.” Displayed data are bandpass-filtered (1–20 Hz) for illustration purposes only.

Here, we present the results of analyzing the ERP data collected in passive session run immediately after the one-trial FM learning session. Аuditory ERPs were recorded to passively presented familiar words and novel native-like word forms, which included those used in the FM session and control (no FM training) items in a fully balanced design. As detailed in the Methods section above, GFP computed over all participants, conditions and electrodes indicated three global peaks at 47, 207 and 323 ms after the divergence point, which led us to selecting three intervals for statistical analysis: 22–72, 182–232 and 298–348 ms from the stimulus divergence point. Average ERPs and mean voltage topographic scalp maps for all conditions are shown in Figure 2 (see also Supplementary Figures 2, 3).

#### 3.1.1 22–72 ms time window

Significant main effect of Stimulus Type was found in the right fronto-central electrode cluster (*F*(1, 19) = 7.101; *p* = 0.015; η^2^ = 0.272) indicating negativity increase for familiar words (both trained and untrained) when compared with the unfamiliar word forms (both trained and untrained). No main effects or interactions involving the Learning Session factor were found in the right fronto-central electrode cluster in this window, and no significant main effects or interactions were found in the left fronto-central cluster in this time window.

#### 3.1.2 182–232 ms time window

In the right fronto-central electrode cluster the results indicated a near-significant Stimulus Type x Learning Session interaction (*F*(1, 19) = 3.583; *р* = 0.074; η^2^ = 0.159). *Post hoc* tests revealed this was due to the significant decrease of the response amplitude to the phonologically native FM-trained novel words when compared with the control unfamiliar word forms (*p* = 0.038). No statistically significant differences between familiar words used in FM condition and control ones were found. No significant main effects of Stimulus Type, Learning Session or interactions were found in the left fronto-central cluster for this time window.

#### 3.1.3 298–348 ms time window

At the last peak, a significant main effect of Learning Session (*F*(1, 19) = 4.9676; *p* = 0.044; η^2^ = 0.197) was found in the left fronto-central cluster indicating negativity increase for FM-trained stimuli when compared to control untrained stimuli. No significant main effects of Stimulus Type, Learning Session as well as no interaction effects were found in the right fronto-central cluster for this time window.

### 3.2 sLORETA results

To estimate neural generators of the observed learning-related ERP dynamics, for the selected ROIs, mean current densities were extracted from 182–232 ms time window after the stimulus divergence point, where a significant response amplitude difference was found in the ERP statistical analysis (see section 3.1.2 above). Statistical analysis of these cortical source data revealed a number of effects.

**Figure 3:**
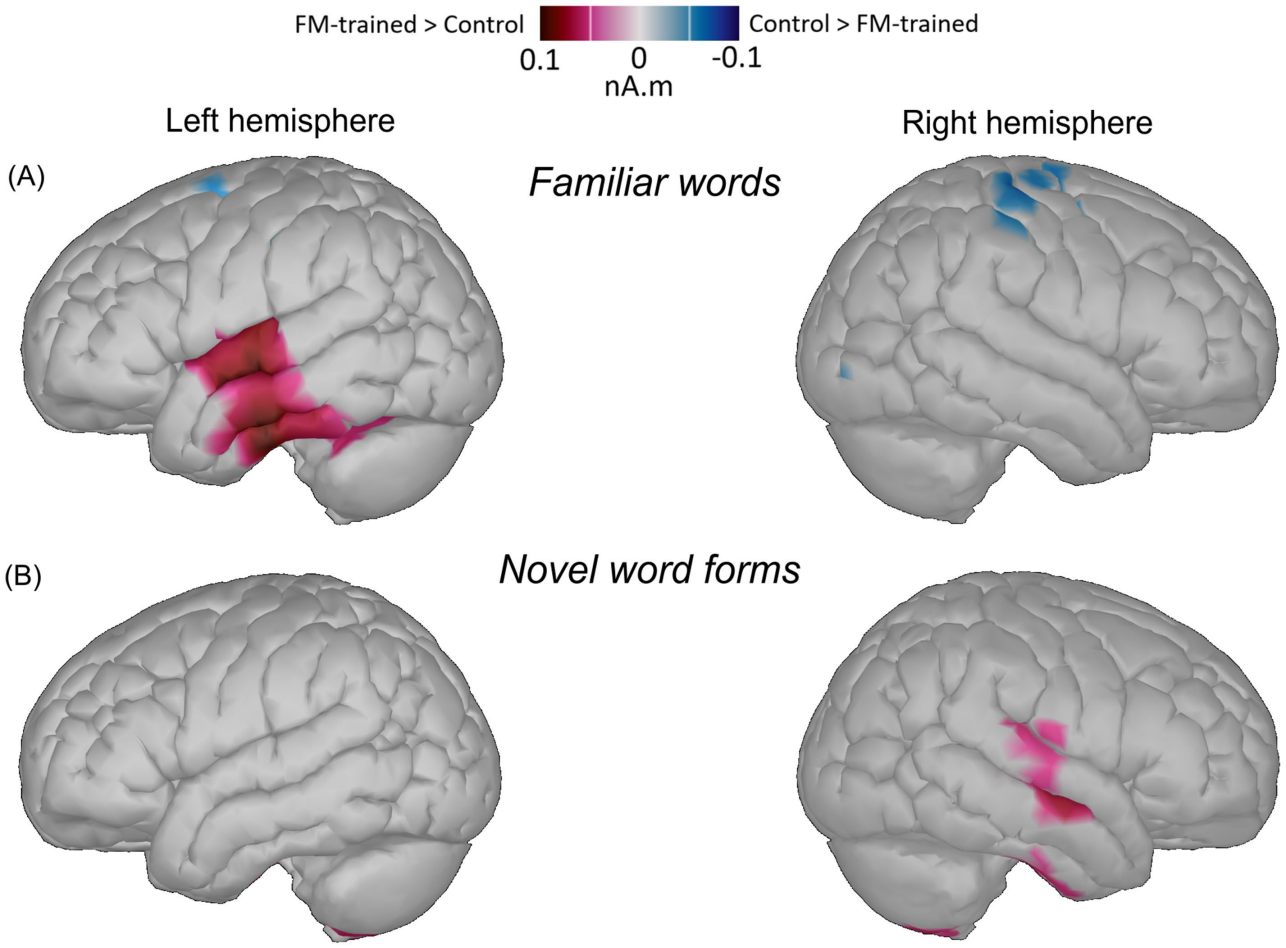
Cortical source activation underlying word learning. Images show differences in averaged sLORETA maps source estimations of the significant response change for familiar words **(A)** and novel word forms **(B)** at 182–232 ms (after stimulus divergence point) elicited in FM and control conditions (FM-trained vs. control condition contrast). Individual source estimations were overlaid onto the age-appropriate MRI templates. Regions of Interest (ROIs) were selected based on the previous investigations on neuroanatomical foundations of language in the temporal lobe (inferior, middle, and superior temporal gyri), temporal pole, inferior frontal, and supramarginal areas as well as their right-hemispheric homologues (see Methods section 2.5 for details).

Namely, in the left hemisphere a near-significant Stimulus Type x Learning Session interaction was found in the frontal portions of both inferior temporal gyrus (fpITG: *F*(1, 19) = 3.685; *p* = 0.07; η^2^ = 0.162) and medial temporal gyrus (fpMTG: *F*(1, 19) = 3.901; *p* = 0.063; η^2^ = 0.170). *Post hoc* tests showed that this was due to the significant decrease of the response to FM-trained familiar words when compared with their familiar counterparts used as control stimuli (*p* = 0.017; *p* = 0.018). Furthermore, a main effect of Learning Session was found in both of these sources (*F*(1, 19) = 3.585; *p* = 0.074; η^2^ = 0.159; *F*(1, 19) = 6.324; *p* = 0.021; η^2^ = 0.250) that indicated a significant decrease in activation for FM-trained stimuli (both familiar words and novel word forms) when compared with untrained stimuli (*p* = 0.014; *p* = 0.007). Also, a significant Stimulus Type effect was revealed in the fpMTG (*F*(1, 19) = 4.673; *p* = 0.044; η^2^ = 0.197) that showed activation increase for both trained and untrained pseudowords when compared with the familiar words (both trained and untrained; *p* = 0.009). Furthermore, similar significant main effects of Learning Session (*F*(1, 19) = 5.025; *p* = 0.037; η^2^ = 0.209) and Stimulus Type (*F*(1, 19) = 4.233; *p* = 0.045; η^2^ = 0.196) were found in the frontal portion of the superior temporal gyrus (fpSTG). *Post hoc* test showed response enhancement for the both types of untrained stimuli when compared against both types of FM-trained ones, as well as a response increase for novel word forms (both trained and untrained) when compared to familiar words. Figure 3 provides a graphic illustration of significant source activity contrasts in the left hemisphere.

Several effects were also identified in the right hemisphere sources. A near-significant interaction Stimulus Type x Learning Session (*F*(1, 19) = 3.887; *p* = 0.063; η^2^ = 0.250) was found in the caudal portion of the superior temporal gyrus (cpSTG). Mirroring the ERP analysis above (see 3.1.2 Section), *post hoc* tests revealed this effect was derived from an activation decrease for the FM-trained novel words (*p* = 0.05), while activation elicited by familiar words did not change. Similar to the effects observed above, a near-significant Learning Session effect was found in the right temporal pole (*F*(1, 19) = 3.694; *p* = 0.070; η^2^ = 0.163) indicating a response drop for FM-trained stimuli (both familiar and novel word forms) when compared to untrained stimuli. No significant effects were found for familiar words in the right-hemispheric sources. Significant source activity contrasts found in the right hemisphere are graphically illustrated in Figure 3.

## 4 Discussion

Here, we present electrophysiological evidence of neural correlates of Fast Mapping – the ultra-rapid word-learning mechanism in the developing brain, previously studied mainly using behavioral methods. In the current experiment, we followed the original design of classical FM studies in children and utilized a one-trial presentation of novel item with assigned semantic referent during a short learning procedure (one single-shot exposure), and analyzed the brain’s activity elicited by these newly learnt items in a passive-listening session immediately after the learning block. To study the process of the rapid novel native word acquisition we used familiar and novel word forms with native phonology. Native familiar and novel speech sounds that did not undergo FM procedure were used as control stimuli. Our results indicated that children between ages 5 and 7 demonstrated distinct changes in electrophysiological activity: significant decrease in ERP amplitudes for newly learnt native word forms was found at 182–232 ms time window (after stimulus divergence point, when each stimulus could be uniquely identified), with no similar changes for familiar native words used in the same experimental conditions as control stimuli. This decrease in neural activity is most likely linked to the formation of novel word representations as a result of the single-trial semantic learning exposure. These results are in line with previous EEG and fMRI adult studies (Mestres-Missé et al., 2007, 2008), indicating that the formation of neural circuits corresponding to new lexical elements of the mother tongue occurs almost instantaneously and is especially efficient when a semantic referent is available during the acquisition of a new vocabulary item. We will briefly discuss these main findings below.

First, the early timing of this change in brain response to meaningful newly learnt words at ~ 200 ms after stimulus divergence point suggests extremely rapid processing of not only lexical (access of surface form) but also semantic (meaning access) information. This finding indicates that, in the developing brain, cortical processes of both lexical and semantic access may commence early-on and may unfold simultaneously within this relatively early time frame.

As commonly posited, lexical processing starts from word onset, and in this sense, the latencies observed in the current study broadly fall within the classic N400 range. However, it is important to note that, despite a large body of research (Fromkin, 1971; Dell, 1986; Huettig and McQueen, 2007) there is no single, universal psycholinguistic model of spoken language processing. Early serial, cascaded models postulated a sequence of processing steps that start at different times, while contemporary neurophysiological studies (for example mismatch negativity-based ones) suggest that all types of linguistic information (lexical, syntactic, semantic) are accessed and processed near-simultaneously (within 200 ms), shortly after stimulus information allows for identification of the critical lexical items (Pulvermuller and Shtyrov, 2006). This rapid access may be followed by later language-related processes that are engaged in second-order computations and reprocessing the information accessed (Pulvermuller and Shtyrov, 2006) and that may underlie the classical language potentials (for example, the N400 and P600) (Friederici, 2002).

The second major finding is that such a learning-related neural dynamics was registered for new items presented audially in just one single trial. That confirms earlier behavioral findings that the child’s brain is capable to form novel word representations from a single instance. Importantly, ERP changes observed here differed in several aspects from the previous investigations of single-shot learning in adults. In our study, the earliest learning-related activity was registered around 200 ms, while previous studies that utilized a traditional contextual task in visual modality reported a classical N400 effect found in a broad 250–500 ms time window for target sentence-final nouns presented in strongly constraining sentence contexts (Borovsky et al., 2010, 2013). The divergence in response latency between these studies could be related (apart from the visual vs. auditory modality) to key differences in paradigms: while using complete sentences entails contextual integration (which might take longer time), the single-word presentation used in the current study likely prompts access for that single item only in the mental lexicon, without the need to integrate it with the preceding context, thereby taking less time.

Another explanation of the observed «early» N400 latency could be driven from the prediction-related habituation framework. Predictive coding theory (Rao and Ballard, 1999; Friston, 2009) proposes that the brain has unique capacity to predict future events on the basis of probabilistic inference and compare these predictions to actual sensory input. In case of match between input and prediction a suppressed neural response is generated, whereas a mismatch elicits a prediction error response, which drives learning and updating of internal models. In language development, this mechanism is supposed to underlie the ability to learn new words and their meanings. Developmental studies reveal that predictive inference, a domain-general developing brain, it must be, may speed up word recognition and support learning of novel words in the child’s brain, including sound to meaning mappings. Predictive coding may mediate learning via attention allocation, which, in turn, may drive the learning of both word forms and the link to their referents. Thus, the interplay between habituation and prediction has important implications for language learning during childhood (Helenius et al., 2014; Ylinen et al., 2017; Reuter et al., 2019; Knowland et al., 2022).

Importantly, the current neural dynamics obtained in 5–7 year-old children are a similar to those previously described for adults using a comparable single-shot FM-paradigm: a significant change in neural activation elicited by the newly trained word form at ~200–400 ms after the word onset (Vasilyeva et al., 2019), confirming this tentative explanation. Furthermore, similarly early onset of N400 is well-known in adult studies using single words (Friedrich et al., 2006) when the stimulus identification point is accounted for, as also done here. These results also confirm the functional significance of the N400 in general and of its early phase in particular, as reflecting the neural efforts involved in lexicosemantic access and search in the mental lexicon. More generally, the current findings can be considered as the first electrophysiological evidence of the remarkably fast word-learning mechanism that exists in the developing brain and operates by means of single-shot semantic mapping.

The nature of the neurophysiological dissociation of meaningful newly learnt words and meaningless pseudowords in the current study may be explained in terms of the Cohort Model of spoken word recognition (Marslen-Wilson, 1984, 1987; Gaskell and Marslen-Wilson, 2002). According to this model, the word recognition processes take place in three stages: those of lexical access, selection and integration. Importantly, it implies an interplay between automatic bottom-up and high-order top-down processes that defines the timeframe at which contextual information contributes to word recognition processes. So, at the initial stage, when speech stimulus is heard (e.g., the initial phonemes */va/* in *vata,* as in our study) this triggers an ongoing process of rapid and partial activation of representations of all multiple word candidates that match the incoming speech signal (word-initial cohort), and a competition between them. For instance, in addition to the stimulus *vata* (cotton wool) in the current design, also *vaza* (vase), *vaksa* (wax), *vakcina* (vaccine), etc. would be partially activated. Parallel processes of activation/competition persist until the critical point when the target word becomes maximally differentiated from all other cohort candidates (i.e., the uniqueness point), while those competitors that mismatch the unfolding speech input are filtered out. It is proposed that the gradual decrease in competition results in boosting of the semantic representation of the target word (Hagoort and Brown, 2000; Kocagoncu et al., 2017). Specifically, in our study it was the second syllable’s initial stop-consonant that determined both the status of the entire stimulus as either a well-known familiar word or a newly learnt one or as a meaningless previous unknown word form. Thus, the observed early N400 effect might reflect the intermediate lexical selection process, when form-based and content-based informations are combined to select the appropriate word and enable the specific word representation to become fully accessed. This, once the possible competitors have been ruled out and their respective activation extinguished, results in the drop of ERP amplitude. Otherwise, the search is continued, with the persistent effort resulting in stronger N400. Notably, however, given the structural and functional specificity of the developing brain, psycholinguistic models designed exclusively for mature adult brain may not necessarily be completely applicable to the child’s brain; these issues therefore require further detailed research.

Indeed, for a broader explanation of the current results, two compatible accounts may be considered, with one explaining the speech signal-driven temporal dynamics of lexico-semantic traces (given above) and the other – specifying the grounded nature of semantic acquisition. Embodied theories of grounded semantics posit that word meaning acquisition leads to the emergence of associative link between the symbol (word form representation emerging as neuronal memory circuit in perisylvian areas) and the corresponding semantic information derived from sensory input and the motor system (Pulvermüller and Fadiga, 2010; Kiefer and Pulvermüller, 2012; Pulvermüller, 2013; Garagnani and Pulvermuller, 2016). That is, acquisition of semantic knowledge is based on its grounding in the real world, and that is exactly what was promoted by the present learning setup, when novel word meaning was grounded in the association between the acoustic/phonological word form and the physical experience of interaction with the object it referred to.

The other interesting and somewhat paradoxical result is that, for the newly learnt words, significant changes in neural dynamics were predominantly observed in the right hemisphere (RH). While the left-hemispheric (LH) involvement as such cannot be excluded by the present data, the statistical analysis of both the ERP amplitudes and the source activations indicated that the presently reported statistically significant learnt-unlearnt contrast is RH-based and appears to be underpinned by activity decrease in the right STG. These findings are in line with a growing number of studies that show active involvement of the RH in various aspects of language function (albeit in more complex ways than that of the LH), ranging from paralinguistic and prosodic feature processing, to comprehension of metaphors and connotations, to semantic processing and word meaning acquisition (Jung-Beeman, 2005; Lindell, 2006; Federmeier et al., 2008; Passeri et al., 2015; Ulanov et al., 2023). Some studies also reported increased activation of RH during higher-level language tasks, such as deriving inferences (Kircher et al., 2001; Mason and Just, 2004), detecting story inconsistencies (Ferstl et al., 2005), correction of grammatical errors (Meyer et al., 2000), or language processing in unfavorable conditions (Shtyrov et al., 1999; Liikkanen et al., 2007), suggesting RH active engagement in supporting resource-demanding neurolinguistic processes. Furthermore, RH homologues of LH language areas have been specifically demonstrated to be involved in the acquisition of novel word forms (Paulesu et al., 2009; Vigneau et al., 2011) which may indicate the recruitment of additional (e.g., attentional or working memory) processes required during the processing of novel items (Vigneau et al., 2011), and is fully in line with the present findings.

Crucially, several comparative functional magnetic resonance imaging (fMRI) studies have shown that children, in contrast to adults, exhibited an overall broader activation during on-line language processing, demonstrating less left-lateralized language networks with stronger involvement of the RH. For instance, studies on auditory sentence comprehension revealed that the magnitude of the right frontal cortex activation was stronger in the youngest group of children (4–6 years old) and exhibited an age-related decline, with young adults showing still present but relatively weaker language activity in the homotopic right frontal and temporal regions (Olulade et al., 2020; Martin et al., 2022). Research data on semantic processing in school-age children demonstrated bilateral activation in the temporal (BA 22) and in the inferior frontal (BA 47/45) gyri; the bilateral nature of this activation was suggested to signify more efficient access lexical-semantic representations in the developing brain (Chou et al., 2006). Another study reported bilateral activity increase during semantic violation task in children, proposing that elevated RH activation reflects higher processing demands (Brauer and Friederici, 2007). These previous results suggesting enhanced RH involvement in language processing in young children (which gives way to LH dominance with maturation) are fully in line with the present finding of the right temporal cortex’s significant contribution to word acquisition.

It is worth noting, however, that although the importance of functional interplay between the two hemispheres in normal language processing is well established, the matter of the relative involvement of the RH and LH hemispheres in the formation of early word representations and acquisition of new semantics remains unclear. These issues had been addressed in a number of neuropsychological and electrophysiological studies (for reviews, see Bookheimer, 2002; Lindell, 2006; Federmeier et al., 2008; Vigneau et al., 2011; Wlotko and Federmeier, 2013; and also see Koivisto and Laine, 2000; Grose-Fifer and Deacon, 2004; Bouaffre and Faita-Ainseba, 2007; Ries et al., 2016). According to the prominent and long-standing «fine-coarse coding theory» (FCT; Beeman et al., 1994; Bowden and Beeman, 1998; Jung-Beeman, 2005), the contributions of RH and LH hemispheres at the stage of initial semantic mapping are qualitatively different. Investigations of semantic priming using split visual field paradigm posited that LH provides «fine» semantic coding, activating a strong and focused semantic fields of closely related word meanings or semantic features. RH, on the other hand, carries out a relatively «coarse» coding and maintains activation of large and diffuse semantic fields (that may overlap and sum up) containing multiple alternative or distantly related or unusual meanings (Beeman et al., 1994; Chiarello et al., 2003; Deacon et al., 2004; Grose-Fifer and Deacon, 2004; Jung-Beeman, 2005; Faust, 2012). This «coarse» coding might be what is provided by the developing brain’s right hemisphere after just a single encounter with the new item; it may well be that, with more exposure and strengthening of the lexicosemantic representation, the LH starts playing a more expressed role.

Indeed, given the prolonged and gradual nature of word learning, fast mapping *per se* may be defined as the very first, initial step in constructing the meaning of a new word (Carey and Bartlett, 1978; Carey, 2010). A more precise understanding of the word meaning is likely to be acquired through a slower process, the so-called «extended mapping», involving multiple encounters with or uses of a word in a variety of contexts (Schwanenflugel et al., 1997; Gaskell and Dumay, 2003; Leach and Samuel, 2007; Carey, 2010; Swingley, 2010; Christ and Wang, 2011). That said, the exact hemispheric contributions in early word representations may be shaped by context and time course, changing with the ongoing experience of using the word during repeated exposures, and this knowledge may be differentially organized and differentially processed by the hemispheres. Several studies have claimed that at the initial stage novel word mappings may rely on RH (providing a more «broad» meaning activation). Subsequently, prolonged experience with the word may be resulted in a stronger categorical semantic activation (depicting «fine-grained» aspects of semantic knowledge) predominantly supported by LH (Dagenbach et al., 1990; Shore and Durso, 1990; Durso and Shore, 1991; Ince and Christman, 2002). This hypothesis had been confirmed in a contextual single-trial word learning study in adults (Borovsky et al., 2013), which showed N400 priming effects only in the RH, with no significant effects in LH. Moreover, some fMRI studies have also associated the right temporal lobe with the fast-mapping phenomenon (Atir-Sharon et al., 2015; Merhav et al., 2015), consistent with the current results. Thus, we can propose that, in the developing brain, the RH may be actively recruited during rapid native word learning, contributing at least to the initial stage of novel semantic mappings. These initial word representations established after a single FM trial potentially have diffuse, «coarse» structure and may be highly dependent on RH for further incorporation into the mental lexicon. However, this suggestion should be treated with due caution as further research is still needed which can address these issues using repetitive tests on multiple days at different time delays from the learning session as well as in different age groups.

In sum, the results of the current study suggest that the developing brain could distinguish familiar and unfamiliar verbal stimuli as early as ~200 ms after the presentation of acoustic information allows for stimulus identification, indicating extremely rapid lexicosemantic processing. Moreover, rapid changes in the brain dynamics are registered for new items presented audially in just one single trial in conjunction with a referent object, indicating that the developing brain is capable of learning words from a single instance, provided that the context allows for unambigious “fast mapping” between the word and the object it denotes. The observed neurophysiological dissociation at ~200 ms could be considered as the distinct index of rapid lexicosemantic processing of single words in the developing brain during a single-shot semantic learning that has not so far been reported in the literature. Moreover, the results indicate that such newly established word representations are supported by right-hemispheric temporal cortical areas. To conclude, our findings provide unequivocal electrophysiological evidence of the remarkably fast mechanism of native word acquisition in the developing brain that promotes rapid integration of novel word representations into neocortical lexicosemantic networks. Further research is needed to uncover the underpinnings of this extremely efficient learning mechanism, to generalize current findings to larger stimulus groups and other aspects of word meaning, to scrutinize the interplay between the immediate and longer-term stages of early word learning, as well to investigate FM-learning effects at both neural and behavioral levels in various experimental groups and clinical populations.

## Data Availability

The raw data supporting the conclusions of this article will be made available by the authors, without undue reservation.

## Footnotes

^1^Behavioral and neurophysiological studies provide evidence that at least two distinct neurocognitive strategies may be involved in acquisition of novel words: context-dependent inference-driven learning, or *fast mapping* (FM), and instruction-based *explicit encoding* (EE) (for review, see Shtyrov et al., 2019). EE typically takes place, e.g., in classroom settings, during teacher-student interaction, when the learnt item is repetitively presented through a direct instruction, in a clear and explicit manner. In contrast, FM normally takes place in a natural environmental setting, during routine communicative interactions (e.g., parent–child), in a familiar context that facilitates new word learning. FM is a learning strategy that relies on contextual inference, when the meaning of the new item is inferred incidentally by excluding other, familiar ones. A cognitive reasoning process, disjunctive syllogism («A or B? Not A, therefore B»), providing the association between the unfamiliar object and the novel label, is believed to support this process (Halberda, 2006). FM is believed to require just a few exposures of novel items (with claims of even single-shot learning).

^2^In Russian Federation, in accordance with the Federal State Educational Standard, education of children in primary schools begins when they reach the age of 6.5 in the absence of any health-related contraindications but not later than the age of 8.

^3^ https://brainsuite.org/
