# Supplementary material for: Neurophysiological evidence of single-shot semantic mapping in the developing brain
Source: Front Hum Neurosci. 2025 Jul 30;19:1533833. doi: 10.3389/fnhum.2025.1533833 (PMC12344786; doi:10.3389/fnhum.2025.1533833)
Supplement: Supplementary file 1 [file Data_Sheet_1.pdf]

Supplementary Material

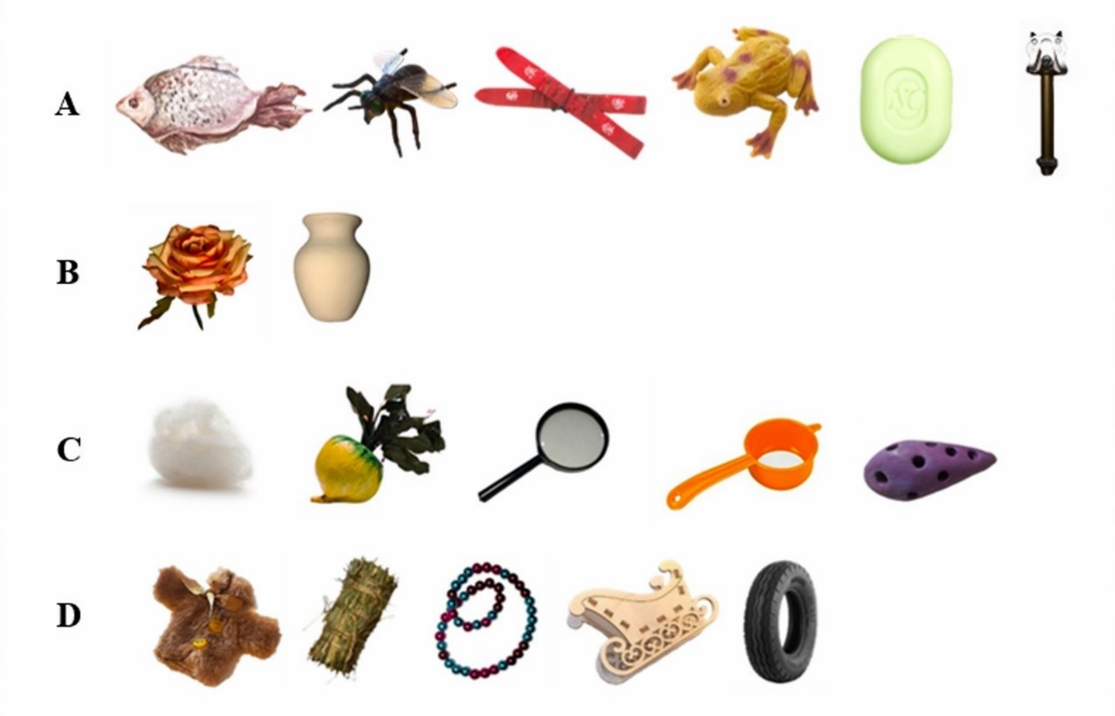

Supplementary Figure 1. Stimuli set from the practice, learning and EEG sessions

(A). In practice session five trials with «familiar word – familiar object» pairs and one trial with an «unknown word – unknown object» pair were used. Five real objects from the practice trials represented familiar items and corresponded to the acoustically presented familiar native words: *ryba (fish), muha (fly), lyzhi (skis), zhaba (bufo), mylo (soap)*. One unknown object from practice trial represented unfamiliar item and corresponded to the acoustically presented novel word form: *muba.*

(B), (C). In FM learning session four trials were used: two additional familiarization trials with «familiar word – familiar object» pairs and two FM trials: one trial included «familiar word – familiar object» pair and the other included the «novel word form - novel object» pair.

(B) Two real objects from the two familiarization trials of the learning session represented familiar items and corresponded to the acoustically presented familiar native words: *rosa (rose), vasa (vase).*

(C) Four real objects from the FM trials of the learning session represented familiar items and corresponded to the acoustically presented familiar native words: *vata (cotton wool), repa (turnip), lupa (magnifying glass), sita (sieve).* One unknown object from the FM trials of the learning session represented unfamiliar item and corresponded to the one of the acoustically presented novel word form: *vapa, reta, luta, sipa.*

(D) Five real objects represented familiar context from the FM trials of the learning session: *shuba (fur coat), seno (hay), busi (beads), sani (sleigh), shina (tire).*

The acoustic stimuli set from the passive EEG session included the following stimuli types: two stimuli used in the FM learning session (one familiar word and one novel word form) and two control untrained stimuli (one familiar word and one novel word form). The tokens were fully rotated and counterbalanced across the sample.

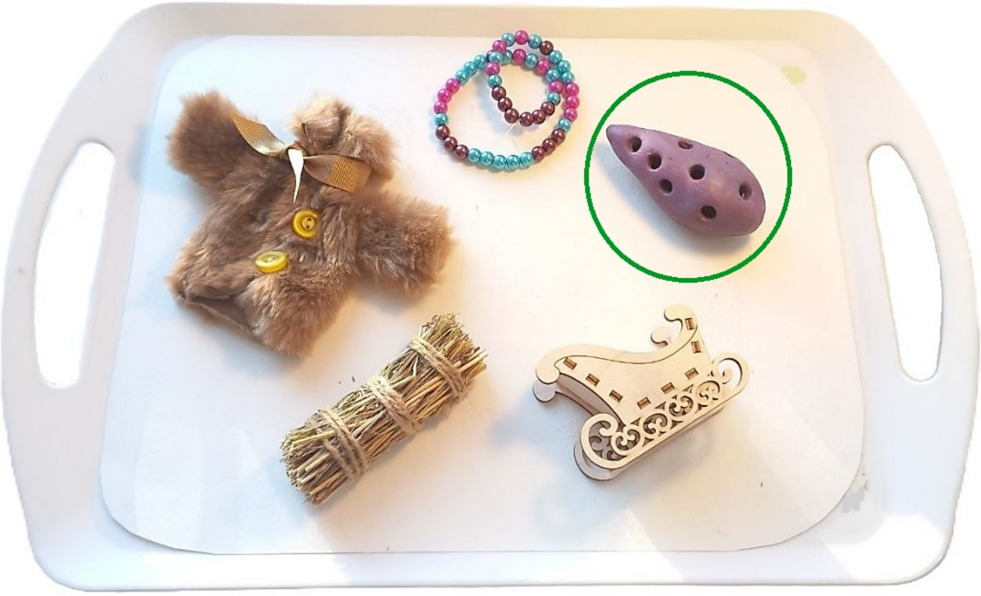

Supplementary Figure 2. Photo of the tray demonstrating the FM trial from the learning session. The target (unknown) object is displayed beside the four other familiar objects *(busi (beads), shuba (fur coat), seno (hay), and sani (sleigh)*. The unknown object is highlighted by a green circle.

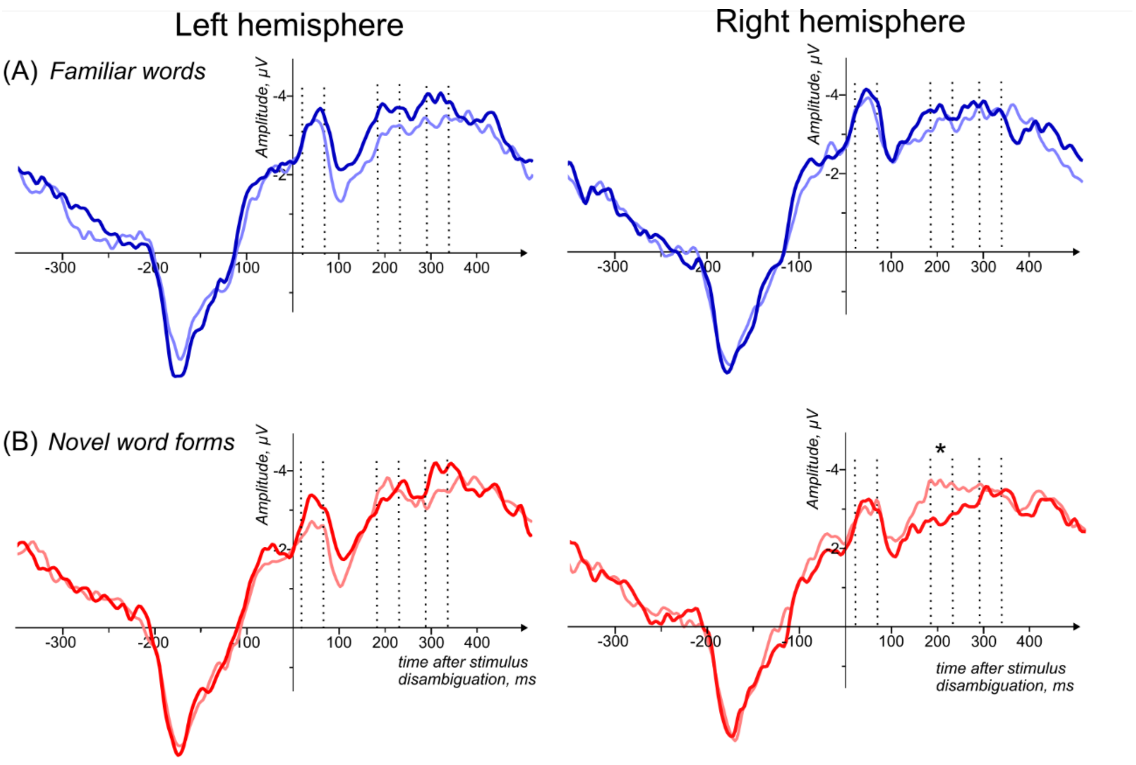

Supplementary Figure 3. Average ERPs in response to familiar words and native-like novel word forms for FM-trained and untrained control conditions at the left and right fronto-central clusters.

(A) Average ERPs in response to familiar words. (B) Average ERPs in response to novel word forms. Negativity is plotted up. ERPs are time-locked to the word divergence point, i.e. the critical second syllable onset after which the stimulus (familiar word vs. novel word form) could be fully identified (the word divergence point corresponds to the zero point on the y-axis). Vertical dotted lines indicate three time intervals (22-72; 182-232; 293-348 ms) taken for the statistical analysis. *Asterisk denote statistical significance at $p < 0.05$ for 182-232 ms time window at the right fronto-central cluster. No significant response changes were found in the FCL.

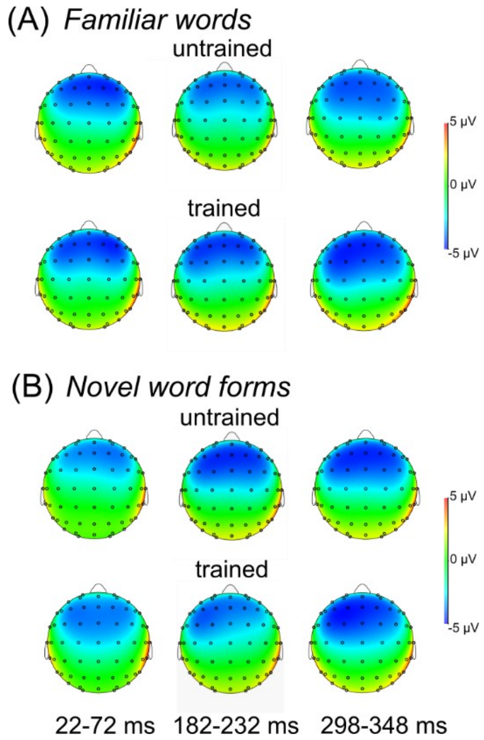

Supplementary Figure 4. Mean voltage topographic scalp maps in response to familiar words (A) and native-like novel word forms (B) for FM-trained and untrained control conditions for three time intervals (22-72; 182-232; 293-348 ms).

Supplementary Table 1. Stimuli frequency of occurrence

words	ipm	D	pseudowords
vata (wadding)	8.2	91	vapa
sita (sifter)	2.6	86	sipa
lupa (loupe)	2.9	90	luta
repa (turnip)	3.6	81	reta

Notes. Ipm and Juilland D value dispersion (Juilland, Brodin, & Davidovitch, 1970) are presented according to the National Corpus of the Russian language (Lyashevskaya & Sharov, 2009; https://www.ruscorpora.ru/) standardised database.

Supplementary Table 2. Analysis of variance (ANOVA) results. ERPs.

SM	Time interval 22-72 ms	Time interval 182-232 ms	Time interval 298-348 ms
Left hemisphere			
Stimulus type (familiar word/ novel word form)	$F(1,19)=1.969$ $p=0.177$ $\eta^2_p=0.094$	$F(1,19)=0.432$ $p=0.519$ $\eta^2_p=0.022$	$F(1,19)=0.687$ $p=0.417$ $\eta^2_p=0.035$
Learning Session (untrained word form/trained word form)	$F(1,19)=2.144$ $p=0.159$ $\eta^2_p=0.101$	$F(1,19)=0.511$ $p=0.483$ $\eta^2_p=0.026$	$F(1,19)=4.676$ $p=0.044$* $\eta^2_p=0.197$
Stimulus type x Learning Session	$F(1,19)=0.316$ $p=0.581$ $\eta^2_p=0.016$	$F(1,19)=0.607$ $p=0.445$ $\eta^2_p=0.031$	$F(1,19)=0.028$ $p=0.868$ $\eta^2_p=0.001$
Right hemisphere			
Stimulus type (familiar word/ novel word form)	$F(1,19)=7.101$ $p=0.015$* $\eta^2_p=0.272$	$F(1,19)=0.345$ $p=0.564$ $\eta^2_p=0.018$	$F(1,19)=0.005$ $p=0.947$ $\eta^2_p=0.001$
Learning Session (untrained word form/trained word form)	$F(1,19)=0.311$ $p=0.584$ $\eta^2_p=0.016$	$F(1,19)=1.780$ $p=0.198$ $\eta^2_p=0.086$	$F(1,19)=0.042$ $p=0.839$ $\eta^2_p=0.002$
Stimulus type x Learning Session	$F(1,19)=0.085$ $p=0.774$ $\eta^2_p=0.004$	$F(1,19)=3.583$ $p=0.074^{\#}$ $\eta^2_p=0.159$	$F(1,19)=0.002$ $p=0.966$ $\eta^2_p=0.001$

*Analysis included data from left and right fronto-central clusters, with factors Stimulus type (familiar word/novel word form) and Learning Session (untrained word form/trained word form); *p < 0.05; # 0.05<p < 0.1.*

Supplementary Table 3. Analysis of variance (ANOVA) descriptive statistics. ERPs.

**Time interval 22-72 ms**				
Stimulus Type	N	Mean	SD	SE
Left hemisphere				
Untrained novel word form	20	-2.55	1.80	0.402
Untrained familiar word	20	-3.03	1.59	0.357
Trained novel word form	20	-3.11	2.08	0.465
Trained familiar word	20	-3.27	1.57	0.351
Right hemisphere				
Untrained novel word form	20	-2.88	2.13	0.475
Untrained familiar word	20	-3.45	1.89	0.423
Trained novel word form	20	-2.99	1.88	0.420
Trained familiar word	20	-3.70	2.52	0.563
**Time interval 182-232 ms**				
Left hemisphere				
Untrained novel word form	20	-3.61	1.54	0.345
Untrained familiar word	20	-3.12	1.95	0.437
Trained novel word form	20	-3.43	1.76	0.393
Trained familiar word	20	-3.57	1.62	0.362
Right hemisphere				
Untrained novel word form	20	-3.50	1.50	0.335
Untrained familiar word	20	-3.15	1.56	0.350
Trained novel word form	20	-2.71	1.14	0.255
Trained familiar word	20	-3.28	1.61	0.360
**Time interval 298-348 ms**				
Left hemisphere				
Untrained novel word form	20	-3.51	1.75	0.392
Untrained familiar word	20	-3.34	1.61	0.359
Trained novel word form	20	-4.07	1.89	0.422
Trained familiar word	20	-3.78	1.71	0.382
Right hemisphere				
Untrained novel word form	20	-3.33	1.88	0.421
Untrained familiar word	20	-3.33	2.02	0.453
Trained novel word form	20	-3.40	2.02	0.451
Trained familiar word	20	-3.36	1.65	0.369

Supplementary Table 4. Analysis of variance (ANOVA) results. Loreta.

SM	**Inferior temporal FP**	**Middle temporal CP**	**Middle temporal FP**	**Superior temporal CP**	**Superior temporal FP**	**T pole**
Left hemisphere						
Stimulus type (word/pseudoword)	F(1,19)=1.030<br>p=0.323<br>η^2_p=0.051	F(1,19)=0.405<br>p=0.532<br>η^2_p=0.021	F(1,19)=4.673<br>p=0.044*<br>η^2_p=0.197	F(1,19)=0.003<br>p=0.956<br>η^2_p=0.000	F(1,19)=4.623<br>p=0.045*<br>η^2_p=0.196	F(1,19)=0.066<br>p=0.799<br>η^2_p=0.003
Learning Session (trained/untrained)	F(1,19)=3.585<br>p=0.074#<br>η^2_p=0.159	F(1,19)=2.301<br>p=0.146<br>η^2_p=0.108	F(1,19)=6.324<br>p=0.021*<br>η^2_p=0.250	F(1,19)=0.311<br>p=0.583<br>η^2_p=0.016	F(1,19)=5.025<br>p=0.037*<br>η^2_p=0.209	F(1,19)=0.597<br>p=0.449<br>η^2_p=0.030
Stimulus type x Learning Session	F(1,19)=3.685<br>p=0.070#<br>η^2_p=0.162	F(1,19)=1.336<br>p=0.262<br>η^2_p=0.066	F(1,19)=3.901<br>p=0.063#<br>η^2_p=0.170	F(1,19)=0.045<br>p=0.834<br>η^2_p=0.002	F(1,19)=2.914<br>p=0.104<br>η^2_p=0.133	F(1,19)=0.369<br>p=0.551<br>η^2_p=0.019
Right hemisphere						
Stimulus type (word/pseudoword)	F(1,19)=0.012<br>p=0.914<br>η^2_p=0.001	F(1,19)=0.192<br>p=0.667<br>η^2_p=0.010	F(1,19)=0.012<br>p=0.914<br>η^2_p=0.001	F(1,19)=0.333<br>p=0.571<br>η^2_p=0.017	F(1,19)=0.145<br>p=0.708<br>η^2_p=0.008	F(1,19)=0.212<br>p=0.650<br>η^2_p=0.011
Learning Session (trained/untrained)	F(1,19)=0.106<br>p=0.749<br>η^2_p=0.006	F(1,19)=1.385<br>p=0.254<br>η^2_p=0.068	F(1,19)=1.697<br>p=0.208<br>η^2_p=0.082	F(1,19)=0.933<br>p=0.346<br>η^2_p=0.047	F(1,19)=0.651<br>p=0.430<br>η^2_p=0.033	F(1,19)=3.694<br>p=0.070#<br>η^2_p=0.163
Stimulus type x Learning Session	F(1,19)=0.019<br>p=0.893<br>η^2_p=0.001	F(1,19)=2.490<br>p=0.131<br>η^2_p=0.116	F(1,19)=2.186<br>p=0.156<br>η^2_p=0.103	F(1,19)=3.887<br>p=0.063#<br>η^2_p=0.170	F(1,19)=2.309<br>p=0.145<br>η^2_p=0.108	F(1,19)=1.898<br>p=0.184<br>η^2_p=0.091

*Analysis included data from left and right fronto-central clusters, with factors Stimulus type (word/pseudoword) and Learning Session (trained/untrained). *$p < 0.05$; # $0.05 < p < 0.1$.*

Supplementary Table 5a. Analysis of variance (ANOVA) descriptive statistics. Loreta. Inferior temporal FP

Stimulus Type	N	Mean	SD	SE
Left hemisphere				
Control (untrained) pseudoword	20	0.333	0.132	0.0295
Control (untrained) word	20	0.380	0.176	0.0393
Trained pseudoword	20	0.361	0.143	0.0320
Trained word	20	0.281	0.0984	0.0220
Right hemisphere				
Control (untrained) pseudoword	20	0.298	0.134	0.0299
Control (untrained) word	20	0.299	0.0871	0.0195
Trained pseudoword	20	0.294	0.129	0.0288
Trained word	20	0.289	0.122	0.0273

Supplementary Table 5b. Analysis of variance (ANOVA) descriptive statistics. Loreta. Middle temporal CP

Stimulus Type	N	Mean	SD	SE
Left hemisphere				
Control (untrained) pseudoword	20	0.281	0.114	0.0256
Control (untrained) word	20	0.321	0.139	0.0311
Trained pseudoword	20	0.288	0.0894	0.0200
Trained word	20	0.272	0.143	0.0321

Right hemisphere				
Control (untrained) pseudoword	20	0.381	0.137	0.0307
Control (untrained) word	20	0.334	0.116	0.0259
Trained pseudoword	20	0.316	0.0855	0.0191
Trained word	20	0.349	0.130	0.0290

Supplementary Table 5c. Analysis of variance (ANOVA) descriptive statistics. Loreta. Middle temporal FP

Stimulus Type	N	Mean	SD	SE
Left hemisphere				
Control (untrained) pseudoword	20	0.372	0.125	0.0280
Control (untrained) word	20	0.391	0.153	0.0342
Trained pseudoword	20	0.379	0.115	0.0258
Trained word	20	0.295	0.104	0.0232
Right hemisphere				
Control (untrained) pseudoword	20	0.324	0.116	0.0260
Control (untrained) word	20	0.294	0.0859	0.0192
Trained pseudoword	20	0.267	0.0803	0.0180
Trained word	20	0.301	0.113	0.0252

Supplementary Table 5d. Analysis of variance (ANOVA) descriptive statistics. Loreta. Superior temporal CP

Stimulus Type	N	Mean	SD	SE
Left hemisphere				
Control (untrained) pseudoword	20	0.307	0.134	0.0299
Control (untrained) word	20	0.313	0.188	0.0421
Trained pseudoword	20	0.299	0.0989	0.0221
Trained word	20	0.296	0.157	0.0350
Right hemisphere				
Control (untrained) pseudoword	20	0.414	0.173	0.0387
Control (untrained) word	20	0.353	0.136	0.0304
Trained pseudoword	20	0.340	0.110	0.0247
Trained word	20	0.382	0.149	0.0333

Supplementary Table 5e. Analysis of variance (ANOVA) descriptive statistics. Loreta. Superior temporal FR

Stimulus Type	N	Mean	SD	SE
Left hemisphere				
Control (untrained) pseudoword	20	0.389	0.147	0.0329
Control (untrained) word	20	0.383	0.194	0.0434
Trained pseudoword	20	0.375	0.103	0.0230
Trained word	20	0.293	0.131	0.0292

Right hemisphere				
Control (untrained) pseudoword	20	0.345	0.162	0.0363
Control (untrained) word	20	0.312	0.134	0.0301
Trained pseudoword	20	0.290	0.126	0.0283
Trained word	20	0.338	0.122	0.0272

Supplementary Table 5f. Analysis of variance (ANOVA) descriptive statistics. Loreta. T pole

Stimulus Type	N	Mean	SD	SE
Left hemisphere				
Control (untrained) pseudoword	20	0.283	0.0816	0.0182
Control (untrained) word	20	0.291	0.0866	0.0194
Trained pseudoword	20	0.285	0.0857	0.0192
Trained word	20	0.270	0.0936	0.0209
Right hemisphere				
Control (untrained) pseudoword	20	0.326	0.119	0.0267
Control (untrained) word	20	0.306	0.115	0.0256
Trained pseudoword	20	0.278	0.121	0.0271
Trained word	20	0.313	0.115	0.0257
